# Inter-Finger Variability of SpO_2_ During Hypoxemia and Step Resaturation

**DOI:** 10.3390/healthcare13202648

**Published:** 2025-10-21

**Authors:** Simon Walzel, Veronika Rafl-Huttova, Martin Rozanek, Petr Kudrna, Marian Rybar, Jakub Rafl

**Affiliations:** Department of Biomedical Technology, Faculty of Biomedical Engineering, Czech Technical University in Prague, 27201 Kladno, Czech Republicrafl@fbmi.cvut.cz (J.R.)

**Keywords:** pulse oximetry, desaturation, oximeter, oxygen saturation, hypoxemia, finger sensor

## Abstract

**Background:** Pulse oximetry is a non-invasive method for continuous monitoring of peripheral blood oxygen saturation (SpO_2_) to estimate arterial oxygen saturation. Previous studies suggested that SpO_2_ measurements show variability depending on the particular finger that is used for measurement. To date, no study has compared all fingers for SpO_2_ under hypoxemia and during continuous simultaneous monitoring with randomization of finger sensor placement. **Objectives**: The aim of this study was to assess the inter-finger variability of SpO_2_ values during sequential desaturation and step resaturation. **Methods**: Forty-three out of forty-five healthy participants (age 23.0 ± 1.8 years, BMI 24.0 ± 4.4 kg·m^–2^) completed the experimental assessment with short-term induced hypoxemia by consecutive inhalation of three prepared gas mixtures with reduced oxygen concentrations (14%, 12%, and 10%). SpO_2_ was measured continuously with the Masimo Radical-97 (Masimo Corp., Irvine, CA, USA) pulse oximeters. **Results**: The SpO_2_ measured on the thumb was lower than all other fingers by 0.6% to 0.7% SpO_2_, a systematic difference that is less than the clinically accepted accuracy of oximeters. No difference in SpO_2_ dynamics was found between any of the fingers during step resaturation. **Conclusions**: A systematic difference in measured SpO_2_ exists between the thumb and the other fingers during desaturation, which should be considered at least as well as the impact of the performance of a particular oximeter, sensor placement or anatomical variability.

## 1. Introduction

Measurement of peripheral blood oxygen saturation (SpO_2_) by pulse oximeters using finger sensors is a common method for continuous monitoring of patients’ health condition. The method is used to rapidly estimate the oxygen saturation of arterial blood and the reliability and accuracy of measurement is essential in acute and intensive care conditions [1]. Besides an incorrect sensor placement, the accuracy of pulse oximeters can be decreased by artificial or varnished nails [2,3], skin pigmentation [4,5], lighting and movement artifacts [6], elevated carboxyhemoglobin or methemoglobin levels, the presence of intravascular dyes, elevated bilirubin levels, severe anemia, and venous pulsation [7]. Problems with accuracy can also occur at low perfusion index (PI) [8,9].

Significant differences were also found between pulse oximeters from different manufacturers in terms of accuracy [10] and early detection of hypoxemia [11,12], which may be important not only in intensive care units, but also in experiments where reduced SpO_2_ values are used as an endpoint and thus may significantly affect the duration of the experiment, such as breathing in simulated avalanche snow [13,14,15]. However, it was assumed in these studies that there were no differences in SpO_2_ between individual fingers.

Based on a questionnaire survey, it was found that 80% of healthcare professionals place sensors of pulse oximeters on the index finger [16]. According to the user’s instructions for the Radical-7 monitor of oxygenation (Masimo Corporation, Irvine, CA, USA) [7], a non-dominant ring or middle finger should be selected for SpO_2_ measurement. The only relevant reason found in the current literature for selecting a specific finger for SpO_2_ monitoring was mentioned in the study of Mizukoshi et al. [16] where the middle finger was recommended for SpO_2_ monitoring based on the highest measured PI. Basaranoglu et al. [17] measured the highest SpO_2_ values on the right thumb and the right middle finger, and therefore they recommended to do measurements on these two fingers. Sur et al. [18] measured the highest SpO_2_ values on both middle fingers and the lowest SpO_2_ values on both little fingers. No statistically significant difference in SpO_2_ values between corresponding fingers of the dominant and non-dominant hand was found. In the study of Juliana et al. [19] the highest mean SpO_2_ values were measured on the earlobe and then on the right ring and middle finger. The lowest values were measured on both little fingers. In summary, it was suggested that the measured SpO_2_ values differ between individual fingers [16,17,18,19]. However, these hypotheses have not yet been sufficiently verified, as the measurements were performed only at physiological SpO_2_ values and not taken on individual fingers simultaneously and by identical type of pulse oximeter.

The aim of this study was to assess the inter-finger variability of SpO_2_ values during sequential desaturation and fast resaturation in a healthy person during short-term induced hypoxemia.

## 2. Materials and Methods

The prospective interventional single-arm study was approved by the Institutional Review Board of the Faculty of Biomedical Engineering of the Czech Technical University in Prague (Act No. C24/2022). All participants read and signed the informed consent. The study has been registered with ClinicalTrials.gov (NCT05681637) on 10 December 2022 and released and posted on 12 January 2023.

### 2.1. Subjects

The study involved 45 healthy Caucasian participants (24 males, 21 females) aged between 20 and 26 years, with Fitzpatrick skin tones I–III; the group characteristics are shown in Table 1. Participants were required to refrain from using nail polish or artificial nails. None of the participants were excluded from the study due to cardiovascular or respiratory disease, pregnancy, diabetes, ongoing acute illness, or any upper limb or hand injuries that could impact peripheral perfusion.

### 2.2. Experimental Setup and Protocol

**Figure 1:**
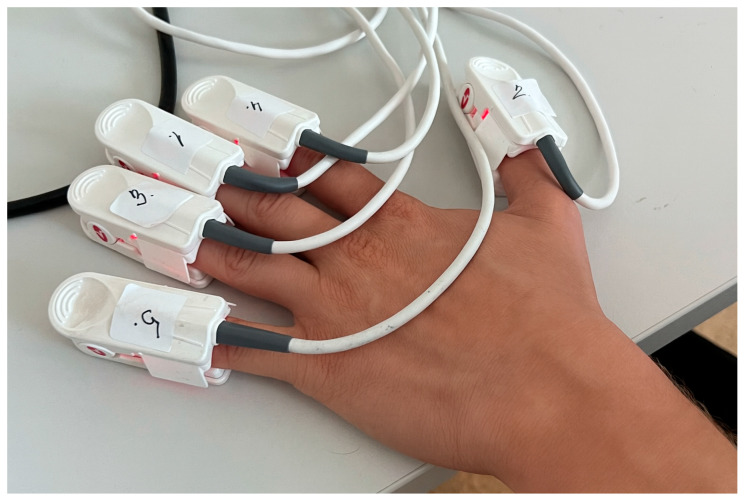
Placement of finger sensors. Masimo Radical-97 (LNCS DCI) sensors were randomly assigned and placed on each finger of the left hand. The participant was seated with the left hand resting still on the table, wrist and palm facing down.

Participants were instructed to rest for a minimum of 30 min before the experimental assessment. Upon arrival at the laboratory, the participants were seated in a comfortable position with their left hand placed on the table in front of them, as in Basaranoglu et al. study [11], and were asked to keep their hands still on the table with the wrist and palm facing down, avoiding any movement. Following the measurement of individual finger circumferences, finger sensors (LNCS DCI) of Masimo Radical-97 pulse oximeters (Masimo Corp., Irvine, CA, USA) were randomly assigned and placed on each finger of the left hand, as shown in Figure 1. SpO_2_ and Perfusion Index were measured continuously on all fingers of the left hand throughout the experimental assessment, with the sampling rate set to 2 s with an averaging time of 0.5 Hz for SpO_2_ and PI set to “short”.

Five fixed sets of the Masimo Radical-97 pulse oximeter and an SpO_2_ sensor (the combinations are hereafter referred to as oximeters #1–#5) were used for all measurements. All five fixed sets were verified for their proper functionality on the ProSim 8 Vital Sign and ECG Patient Simulator (Fluke Corporation, Everett, Washington, DC, USA) at all combinations of PI = 0.1%, 1%, and 10%, and SpO_2_ = 97%, 85%, and 70%. No deviations were found for any set for any of the adjusted combinations of PI and SpO_2_. Oximeters #1–#5 were assigned to the participant’s fingers using a randomized block design to ensure that each oximeter was assigned to each finger with equal frequency. The placement was randomized using the Mersenne Twister pseudorandom number generator algorithm in MATLAB (v.9.14.0, R2023a, MathWorks, MA, USA). The experimental setup involved a non-rebreathing respiratory circuit. It allowed the participant to breathe in either a gas mixture with reduced oxygen concentration from the Douglas bag or the surrounding ambient air, and to exhale into the ambient air outside the Douglas bag. An anesthesia mask covering both the mouth and nose was worn by participants during the experimental assessment. The inhaled gas mixtures were monitored continuously using a Datex Ohmeda S/5 patient monitor (Datex-Ohmeda Inc., Madison, WI, USA) with a respiratory sensor D-Lite (Datex-Ohmeda, Madison, WI, USA) placed in the respiratory circuit. A disposable antibacterial filter was used to maintain separation between the participant and the breathing circuit.

Each experimental assessment lasted approximately 12 min and consisted of three phases. During the initial 2-min stabilization phase, participants inhaled the ambient air via the non-rebreathing circuit. This was followed by a 7.5-min sequential desaturation phase, during which participants inhaled three hypoxic gas mixtures (14% O_2_, 12% O_2_, 10% O_2_) consecutively from the Douglas bag under normobaric conditions, each lasting 2.5 min. The oxygen concentrations were reduced to levels corresponding approximately to altitudes of 3200 m (14% O_2_), 4400 m (12% O_2_), and 5800 m (10% O_2_). In the final stabilization phase, participants inhaled ambient air through the breathing circuit until stable readings were obtained, which took approximately 2 min.

During the experimental assessment, continuous video recording captured the participant, all apparatus, and the complete experimental workplace. This served as a backup of the recorded data and measurement protocols. Device synchronization was ensured by setting the same device time and the subsequent verification of the correct synchronization was performed by comparing the heart rate between oximeters on the video recordings.

### 2.3. Data Extraction and Analysis

The methodology of the present study was based on the International Organization for Standardization guideline (ISO 80601-2-61:2017) for in vivo accuracy testing of pulse oximeters. This guideline requires, among others, at least 200 SpO_2_ readings to be taken from at least 10 subjects, with the readings being balanced across the SpO_2_ range of 70–100% [20]. Based on our previous studies and bench testing, we estimated the standard deviation of SpO_2_ measured on individual fingers from the overall mean to be 1.4%. We determined that 45 participants would be needed to detect a mean difference in SpO_2_ between fingers of 2% (accuracy reported by the oximeter manufacturer) with more than 80% power and an alpha of 0.05. We used G*Power 3.1 (Heinrich-Heine-Universität Düsseldorf, Germany) for the power analysis [21].

Continuous SpO_2_ recordings from oximeters #1–#5 were accessed using the Masimo Instrument Configuration Tool software (V1.2.2.4, Masimo Corporation, Irvine, CA, USA) and sorted for each participant and finger. To determine inter-finger and inter-oximeter variability, oximeter readings were analyzed in 10-s intervals during the initial stabilization phase and sequential desaturation, for the first 9 min 30 s of experimental assessment. The linear mixed-effect model was used for SpO_2_ as the response variable, with finger, oximeter, and time as fixed effects and subject as a random effect. Type III analysis of variance (ANOVA) was performed on the model. The *p*-value for post hoc pairwise comparisons was adjusted with Tukey’s honestly significant difference (HSD) method. A statistically significant result was considered when *p* < 0.05. The software used for data processing and statistical analysis included R (v4.3.2; R Core Team 2021, Vienna, Austria), RStudio (v2023.9.0.463), and G*Power (v.9.14.0, R2023a, MathWorks, MA, USA) [22,23].

**Figure 2:**
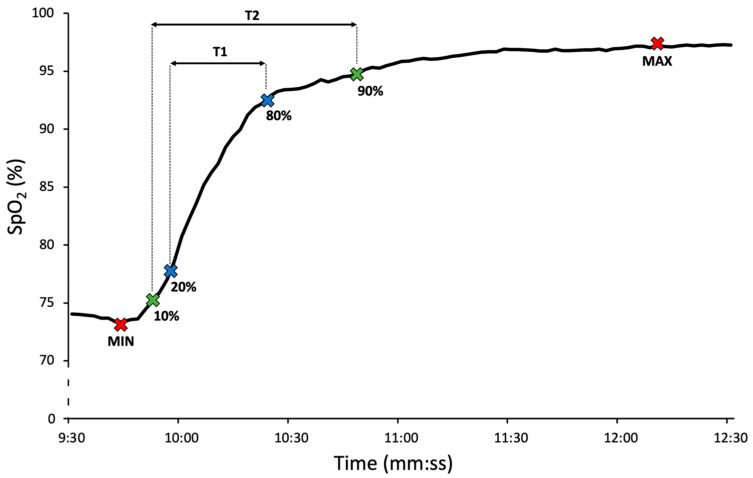
The SpO_2_ response time to step change from breathing the hypoxic mixture to ambient air was assessed by intervals T1 and T2. T1 is the interval between the times when SpO_2_ reaches 20% and 80% of its resaturation amplitude during the final stabilization phase. T2 is the interval between the times when SpO_2_ reaches 10% and 90% of its resaturation amplitude.

The resaturation response time of each finger’s SpO_2_ to a step change from breathing the hypoxic mixture to ambient air was assessed by T1 and T2 intervals which represent the time between 20% and 80%, and 10% and 90%, respectively, of the SpO_2_ amplitude during the final stabilization phase, as depicted in Figure 2. Repeated measures ANOVA was used for each of the three intervals, assuming no significant difference in the dynamic response between the oximeters.

## 3. Results

The study was conducted in February 2023 at the Faculty of Biomedical Engineering in Kladno, Czech Republic, in the Laboratory of Special Equipment for ICU (intensive care unit). Forty-three participants completed the experimental assessment successfully, while two were excluded from the final data analysis due to early termination, resulting in over 12,000 SpO_2_ readings during the initial stabilization phase and the desaturation phase.

The thumb had the highest measured finger circumference with a mean value of 5.9 cm, while the little finger had the lowest with a mean value of 4.3 cm (Table 2). In terms of measured PI, the fingers were ranked similarly, with the highest median value on the thumb at 2.6% and the lowest on the little finger at 1.7%.

**Figure 3:**
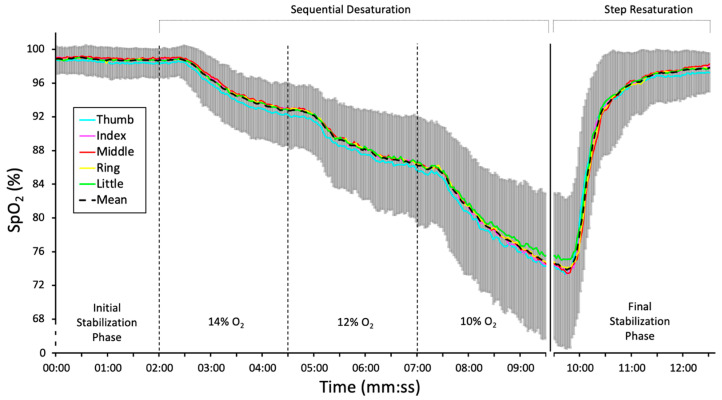
The mean time course of SpO_2_ for each finger during the experimental assessment. Data are presented as mean ± standard deviation across all participants and oximeters.

Figure 3 shows a decrease in SpO_2_ values during the sequential desaturation phase, with the mean SpO_2_ of 74% at the start of the final stabilization phase. SpO_2_ variability between participants increased during the sequential desaturation phase, with a standard deviation of up to 10% SpO_2_. A rapid increase of SpO_2_ back to normal was observed during the final stabilization phase.

**Figure 4:**
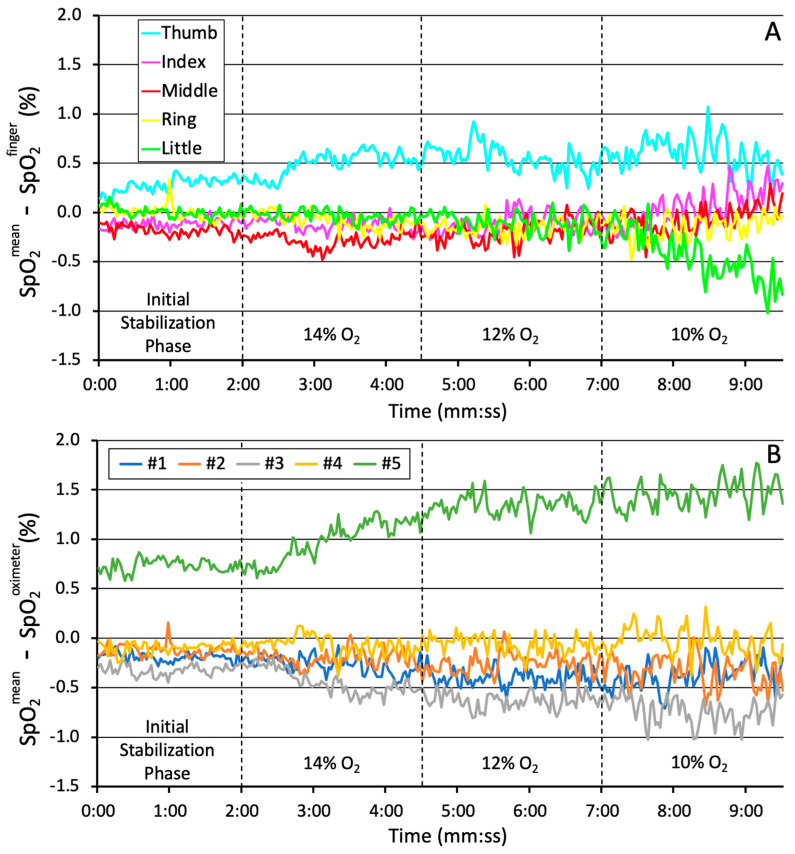
The difference between a finger SpO_2_ and the overall mean SpO_2_ (**A**) and the difference between an oximeter SpO_2_ and the overall mean (**B**) during the initial stabilization and sequential desaturation. #1–#5 indicates the number of the oximeter and SpO_2_ sensor set.

The differences between the overall mean SpO_2_ and the mean SpO_2_ measured at individual fingers or oximeters are demonstrated in Figure 4. During the initial stabilization phase, a difference of approximately 0.3% SpO_2_ was observed between the thumb and other fingers (Figure 4A). This bias increased to about 0.6% SpO_2_ during the sequential desaturation phase. At the end of the desaturation, when the mean SpO_2_ values dropped typically below 80%, the SpO_2_ measured at the little finger raised to almost 1% above the overall mean. Oximeter #5 was notably different from all other oximeters (Figure 4B), with SpO_2_ readings lowered about 0.6% and 1.5% SpO_2_ below the overall mean during the initial stabilization phase and the sequential desaturation phase, respectively.

The analysis of variance found significant effects of finger, oximeter, and time on SpO_2_ (*p* < 0.001) during the initial stabilization and the sequential desaturation. Simultaneous pairwise comparisons of individual fingers indicated that SpO_2_ measurements on the thumb were 0.6–0.7% SpO_2_ lower than on any other finger (*p* < 0.001), as presented in Table 3. The 95% confidence intervals ranged from 0.3% to 1.0% SpO_2_. The post hoc analysis further showed (Table 4) that the average absolute difference between oximeters used in our study ranged from 0.1% (non-significant) up to 1.7% SpO_2_ (*p* < 0.001).

The resaturation response times when inhaling ambient air averaged about 18–19 s (T1) and about 31–33 s (T2) and were slightly longer for the thumb, but no statistically significant difference was found. These results showed high inter-participant variability as indicated by the standard deviation (Table 5).

## 4. Discussion

Our results showed statistically significant differences in SpO_2_ values between the thumb and other fingers, as well as variations between oximeters during the sequential desaturation, when SpO_2_ values decreased across all fingers, with the thumb consistently showing the lowest SpO_2_ readings compared to other fingers. There were no significant differences between other fingers although we observed some deviations from the mean at the little finger at the lower SpO_2_ values. No statistically significant differences in SpO_2_ were found between fingers when reflecting the dynamics of resaturation.

We found that SpO_2_ measurement on the thumb differed systematically during desaturation from all the other fingers by 0.6 to 0.7% SpO_2_, a noticeable but small difference that falls within the expected measurement variability and the tolerance specified by the oximeter manufacturer. The difference in SpO_2_ due to measurement on different fingers was exceeded by measurement variations of individual units of equipment even for the same device of the same manufacturer, both in individual measurements and on average. The oximeters we used differed repeatedly from each other by up to 2% of SpO_2_, but this is consistent with the Root Mean Square Error (RMSE) reported by the manufacturers and required by ISO and Food and Drug Administration (FDA) standards [20,24] for transmittance oximeters.

Our results are not consistent with the prior normoxemic studies conducted only at physiological values of SpO_2_ [17,18,19]. In the study by Basaranoglu et al. [17], the lowest mean SpO_2_ was measured on the left middle finger (97.05 ± 1.94%) and the highest was measured on the right middle finger (98.19 ± 1.20%). The authors partially attributed the differences to hand dominance; however, even the little finger contradicted this pattern, with the left finger measuring higher than the right (97.51% vs. 97.30%). Juliana et al. [18] found that SpO_2_ means ranged from 96.6% on the left little finger to 97.3% on the right middle and ring fingers. The mean between-finger differences were small and within typical device accuracy. In Sur et al. [19], the non-dominant little finger showed the lowest mean SpO_2_ (96.92%), while the dominant middle finger showed the highest (99.08%). However, some fingers had higher mean values on the non-dominant hand than the dominant hand, indicating no consistent dominance effect. Importantly, none of these studies randomized the use of pulse oximeters across measurement sites, even though device-specific bias can influence recorded SpO_2_. In contrast, our protocol involved simultaneous measurements with verified devices during controlled desaturation and resaturation, which allowed us to make direct comparisons between fingers. This methodological difference likely explains the smaller inter-finger differences observed in our study.

We observed no statistically significant differences in the resaturation response time between the fingers in reaction to the step change from breathing the hypoxic mixture to ambient air. The resaturation response time was also evaluated in the studies by Choi et al. [25] to compare finger transmittance and forehead reflectance pulse oximeters and by Trivedi et al. [11] to compare oximeters from different manufacturers. Choi et al. [25] reported 23.2 ± 5.6 s (reflectance) and 28.9 ± 7.6 s (transmission) to reach SpO_2_ = 100% with 100% oxygen, whereas for the index finger in our study resaturation on ambient air yielded T1 = 18.5 ± 14.6 s and T2 = 31.9 ± 22.5 s. A faster resaturation response time detected by Choi et al. was likely caused by the administration of 100% oxygen after a desaturation period and less pronounced desaturation.

PI, indicating perfusion, is a relatively new vital sign, determined from the pulsatile part of the plethysmography curve, with typical values ranging from 0.02% to 20%. A bias between SpO_2_ and arterial oxygen saturation (SaO_2_) was found when PI was less than 2% [26] and this was recently supported by Louie et al. [8] where low PI was associated with increasing mean absolute bias between SpO_2_ and SaO_2_, i.e., the decreased accuracy of pulse oximetry. The highest SpO_2_ values were measured on the little finger, which had the lowest average finger circumference and PI, and the lowest values were measured on the thumb, which had the highest circumference and PI. The differences in PI and circumference between fingers could therefore lead to differences in SpO_2_ measured on different fingers, assuming that there is no real difference in SaO_2_ between fingers, but rather the difference is caused by the measurement equipment and the calculation algorithm. After all, recommendations in most pulse oximeter manuals define maximum finger sizes for correct measurement.

This study had several limitations. First, the study population consisted of only young healthy individuals, and the findings may not fully translate to patients with underlying medical conditions or physiological variations. Second, the experimental assessment did not include the evaluation of SaO_2_ due to its invasive nature, necessitating arterial blood sampling, which would have significantly complicated the experimental procedure and raised safety concerns for the participants. Third, the experimental assessment involved only one type of pulse oximeter with its averaging time preset to 2 s. To generalize the measured results, it is advisable to repeat the experiment with more types of pulse oximeters from various manufacturers. Also, in this work, we only considered a finger clip sensor, which is the most typical scenario for larger children and adults, but not for newborns, for example, where measurements are taken on the foot or wrist and the whole problem is irrelevant. Next, measurements were taken on one hand only. The other hand was reserved for intermittent blood pressure monitoring, in accordance with ISO 80601-2-61 [20], and for communicating with the medical supervisor regarding desaturation tolerance. The physician assessed consciousness using simple arithmetic tasks, and participants indicated their answers using right-hand fingers. Further, we have not found any explanation as to why one set of oximeter with SpO_2_ sensor systematically differed from the others. Apart from verifying the accuracy of PI and SpO_2_ measurements on the simulator, the oximeter and SpO_2_ sensor sets were the same version, and the sensors were cleaned regularly. Another limitation is that all participants were Caucasian with Fitzpatrick skin tones I–III. This may limit the generalizability of the results to populations with darker skin tones, in which pulse oximeter accuracy can decrease, particularly at lower saturations [27]. Next, the study focused on short-term induced hypoxemia, and the results may not reflect SpO_2_ variability in other clinical scenarios. Last, our method of inducing hypoxemia cannot achieve the same levels of desaturation across all participants as demonstrated by increased SpO_2_ variability towards the end of the desaturation phase. This could have affected the dynamics of resaturation that started at different SpO_2_ levels in different participants considering the nonlinear nature of the oxygenation. Still, our main concern was the within-subject variability of finger SpO_2_.

## 5. Conclusions

This was the first experimental study to evaluate the inter-finger variability of SpO_2_ values while suppressing inter-oximeter variability and the sequential measurement effect. A systematic difference in measured SpO_2_ was found between the thumb and the other fingers during desaturation, averaging 0.6–0.7% SpO_2_. This difference appears clinically insignificant relative to the accepted accuracy limits of pulse oximeters (±2–3%). Variability in individual SpO_2_ measurements at different locations and times should be expected due to random fluctuations and various influences such as the performance of a particular oximeter, sensor placement, or anatomical variability. Accordingly, we recommend measuring SpO_2_ on a finger with a reliable perfusion index and avoiding fingers with suboptimal sensor fit. The difference between fingers is one such marginal factor that is usually not substantial, but may need to be considered in specific applications of this technology. No significant difference in SpO_2_ dynamics was found between any of the fingers during step resaturation.

## Figures and Tables

**Table 1:** The demographic characteristics of the study group.

Parameter	All Participants (*N* = 45)
Age (years)	23 ± 2 (20–28)
BMI (kg·m^−2^)	24 ± 4 (19–37)
Heart rate (bpm)	75 ± 13 (48–99)
Systolic pressure (mmHg)	122 ± 10 (107–149)
Diastolic pressure (mmHg)	72 ± 10 (57–88)

The values are presented as mean ± standard deviation and range (minimum–maximum). BMI—Body Mass Index.

**Table 2:** Measured circumferences and perfusion indices of fingers in the study group.

Finger	Circumference (cm)	PI (%)
Thumb	5.9 ± 0.9 (4.5–8.5)	2.6 (3.1)
Index	5.0 ± 0.7 (3.5–7.0)	2.1 (3.0)
Middle	5.1 ± 0.8 (4.0–7.0)	2.5 (3.5)
Ring	4.9 ± 0.8 (4.0–8.0)	2.1 (3.2)
Little	4.3 ± 0.7 (3.0–6.0)	1.7 (2.1)

The circumference values are presented as mean ± standard deviation and range (minimum–maximum) and the PI values are presented as the median and interquartile range.

**Table 3:** Pairwise comparisons of fingers during the initial stabilization and sequential desaturation.

Finger 1	Finger 2	SpO_2_ Mean Difference Finger 1–Finger 2 (%)	Standard. Error (%)	95% Confidence Interval for SpO_2_ Mean Difference		*p*-Value
				Lower Bound	Upper Bound	
Little	Ring	0.07	0.09	−0.18	0.33	0.939
Little	Middle	0.01	0.09	−0.25	0.26	1.000
Little	Index	0.13	0.09	−0.13	0.39	0.642
Little	Thumb	0.70	0.09	0.44	0.96	<0.001 *
Ring	Middle	−0.06	0.09	−0.32	0.19	0.961
Ring	Index	0.06	0.09	−0.20	0.31	0.974
Ring	Thumb	0.63	0.09	0.37	0.88	<0.001 *
Middle	Index	0.12	0.09	−0.14	0.38	0.701
Middle	Thumb	0.69	0.09	0.44	0.95	<0.001 *
Index	Thumb	0.57	0.09	0.31	0.83	<0.001 *

The Adjustment for multiple comparisons: Tukey’s HSD; * Statistical significance (*p* < 0.05).

**Table 4:** Pairwise comparisons of individual oximeters used in the study during the initial stabilization and sequential desaturation.

Device 1	Device 2	SpO_2_ Mean Difference Device 1−Device 2 (%)	Standard. Error (%)	95% Confidence Interval for SpO_2_ Mean Difference		*p*-Value
				Lower Bound	Upper Bound	
Oximeter #1	Oximeter #2	0.06	0.09	−0.20	0.31	0.977
Oximeter #1	Oximeter #3	−0.24	0.09	−0.49	0.02	0.087
Oximeter #1	Oximeter #4	0.26	0.09	0.00	0.52	0.045 *
Oximeter #1	Oximeter #5	1.45	0.09	1.20	1.71	<0.001 *
Oximeter #2	Oximeter #3	−0.29	0.09	−0.55	−0.04	0.016 *
Oximeter #2	Oximeter #4	0.20	0.09	−0.05	0.46	0.188
Oximeter #2	Oximeter #5	1.40	0.09	1.14	1.65	<0.001 *
Oximeter #3	Oximeter #4	0.50	0.09	0.24	0.75	<0.001 *
Oximeter #3	Oximeter #5	1.69	0.09	1.43	1.95	<0.001 *
Oximeter #4	Oximeter #5	1.19	0.09	0.94	1.45	<0.001 *

The Adjustment for multiple comparisons: Tukey’s HSD; * Statistical significance (*p* < 0.05).

**Table 5:** The SpO_2_ response time of each finger to step change from breathing the hypoxic mixture to ambient air.

	Thumb	Index	Middle	Ring	Little	*p*-Value
**T1 (s)**	19.2 ± 15.2	18.5 ± 14.6	17.9 ± 15.1	18.0 ± 14.3	19.1 ± 18.2	0.712
**T2 (s)**	33.3 ± 22.6	31.9 ± 22.5	32.0 ± 21.1	31.1 ± 22.1	32.4 ± 22.9	0.518

The values are presented as mean ± standard deviation. Repeated measures ANOVA was used for the analysis.

## Data Availability

The datasets generated and analyzed during the current study are available in the repository at https://ventilation.fbmi.cvut.cz/data/ (accessed on 22 September 2025).

## Abbreviations

The following abbreviations are used in this manuscript:

SpO_2_	Peripheral blood oxygen saturation
PI	Perfusion index
BMI	Body mass index
ISO	International Organization for Standardization
ANOVA	Analysis of variance
HSD	Honestly significant difference
ICU	Intensive care unit
RMSE	Root mean square error
FDA	Food and Drug Administration
SaO_2_	Arterial oxygen saturation
